# Bayesian experimental design for optimizing medium composition and biomass formation of tobacco BY-2 cell suspension cultures in stirred-tank bioreactors

**DOI:** 10.3389/fbioe.2025.1617319

**Published:** 2025-09-19

**Authors:** Lars Leyendecker, Henrik Nausch, Christian Wergers, Dirk Scheffler, Robert H. Schmitt

**Affiliations:** ^1^ Department for Production Quality, Fraunhofer Institute for Production Technology IPT, Aachen, Germany; ^2^ Department Model-Based Product and Bioprocess Engineering, Fraunhofer Institute for Molecular Biology and Applied Ecology IME, Aachen, Germany; ^3^ Laboratory for Machine Tools and Production Engineering (WZL) of RWTH Aachen University, Aachen, Germany

**Keywords:** biopharmaceuticals, upstream production, batch fermentation, process optimization, Bayesian optimization

## Abstract

**Introduction:**

The plant suspension cell culture Bright Yellow-2 (BY-2) can be an economic platform for producing complex biopharmaceuticals like cytokines and antibodies at small- and medium-scale because of potentially reduced cultivation and purification costs compared to mammalian cells. This is especially relevant for rare diseases. However, the productivity is currently low in terms of biomass formation in a typical batch fermentation. A potential reason might be that the standard BY-2 cultivation medium, as it is used under laboratory shake-flask cultivation conditions, has not yet been comprehensively optimized and tested in industrial bioreactor settings, addressing all four macronutrients relevant for biomass formation (i.e., sucrose, ammonium, nitrate, and phosphate) in parallel. In this article, we therefore propose a multi-variate, multi-objective, and batch-wise Bayesian experimental design (BED) approach for optimally parameterizing macronutrient supply in the cultivation medium, promoting the fresh mass (FM) increase (i.e., growth rate) and final FM (i.e., biomass).

**Methods:**

We performed a sequential and adaptive experimentation utilizing the BED to optimize the cultivation medium in four iterations with four different media each and confirmed the results in two additional experimentation rounds.

**Results:**

Our results show that while nitrate and phosphate can be used to adjust the growth rate (i.e., reaching up to 40 g/L × d FM), it is possible to reduce sucrose and ammonium without impacting the growth rate and only affecting the final biomass yield (i.e., reaching up to 300 g/L FM). Thereby, we improved the overall productivity of biomass formation (i.e., as a ratio between nutrient and FM input and FM output) for this batch fermentation process by 36%.

**Discussion:**

The results demonstrate the advantages of BED to generate new medium compositions (i.e., macronutrient concentrations) by unbiasedly varying the design space. Thereby, the discovery of new process insights (i.e., impact of individual macronutrient concentrations on the growth rate and biomass formation) is facilitated. Moreover, we show that our data-efficient and black-box BED approach represents a promising alternative to traditional design of experiments (DoE) and mechanistic white-box modeling. Established for the plant suspension cell line BY-2, our BED approach might also apply to yeast and mammalian cells.

## 1 Introduction

The growing prosperity and aging society worldwide have led to a constant rise of non-communicable civilization diseases such as diabetes (affecting 30% of the world population), cancer (affecting 1% of the world population), and numerous rare diseases (affecting in total up to 6% of the world population, whereby each individual disease accounts for less than 0.001%). These diseases cannot be efficiently treated with synthetic drugs (small-molecules). Accordingly, there is an increasing demand for recombinant complex biopharmaceuticals like growth factors, metabolic enzymes, and antibodies (biologics) (World Health Organization, 2024). These biopharmaceuticals are predominantly produced in mammalian cell cultures like human embryonic kidney (HEK) or Chinese hamster ovary (CHO). However, due to the high risk of contamination with human viruses (requiring absolute sterility during production and sophisticated purification processing), mammalian cells are only economic at large-scale (e.g., 2,000-L to 20,000-L bioreactors producing kg amounts) but not at small- or medium-scale (e.g., 5-L to 500-L bioreactors providing g amounts), which is why production costs can exceed 150,000 €/g of biopharmaceutical for rare diseases (Kelley, 2009; Hernandez et al., 2018; Puetz and Wurm, 2019; Walsh and Walsh, 2022). In these cases, plant cell cultures (PCCs) might be a vegan alternative (free of any animal components) because human pathogens do not replicate in plant cells, enabling simplified production and purification processes and making this production platform also economic at small- and mid-scale, potentially lowering the costs to 10,000 €/g (Nausch et al., 2023b). For these reasons, a glucocerebrosidase for the treatment of *Morbus Gaucher* and an α-galactosidase for the treatment of *Morbus Fabry* are already produced in plant cells (Protalix, Israel) as approved drugs (Xu et al., 2011; Xu and Zhang, 2014; Santos et al., 2016; Moon et al., 2019; Nausch et al., 2023b).

Among different PCCs like carrot (*Daucus carota*), rice (*Oryza sativa*), and tobacco (*Nicotiana tabacum*), the tobacco cell line Bright Yellow-2 (BY-2) became the prevalent PCC for the production of biopharmaceuticals (Nagata et al., 1992; Kowalczyk et al., 2022; Nagata, 2023). This is because the BY-2 cell line has the highest growth rate among plant cells ( $\mu_{\max}=0.03/h$ corresponding to a doubling time of 25–30 h compared to a $\mu_{\max}=$ 0.001–0.004 or a doubling time of 250–500 h for other PCCs), being similar to HEK and CHO. Moreover, the BY-2 cell line can be grown to high cell densities of up to 600 g/L fresh mass (FM) (corresponding to 18 g/L dry mass (DM)) in 10,000-L to 100,000-L large-scale stirred-tank reactors (STRs), and several operation modes like batch, fed-batch, and semi-continuous fermentation regimes are already established (Hogue et al., 1990; Gao and Lee, 1992; Ho et al., 1995; Fischer et al., 1999; Ullisch, 2012; Ullisch et al., 2012; Reuter et al., 2014; Raven et al., 2015; Häkkinen et al., 2018). Given these features and due to its higher productivity compared to other PCCs in commercial settings, the BY-2 cell line is preferred for industrial applications, particularly for the production of biopharmaceuticals for rare diseases. For example, Protalix recently shifted from carrot to BY-2 cells (Hanania et al., 2017). Nevertheless, PCCs still represent a niche because the yields of 10 mg to 1 g/L tend to be lower than those of 100 mg to 6 g/L, which can be achieved in HEK and CHO by batch and fed-batch fermentation (Kunert and Reinhart, 2016).

Similar to CHO cells (Schinn et al., 2021; Yeo et al., 2022), it has been recently demonstrated that the volumetric productivity of PCCs can be substantially increased by model-based optimization of the cultivation in a typical batch fermentation setting (Nausch et al., 2023a). For example, in an iterative design of experiment (DoE)-based experiment–modeling–optimization workflow, a mechanistic (Monod-type) white-box model was established that linked the nutrient consumption to the biomass formation. The model is based on the macronutrients sucrose, ammonium, nitrate, and phosphate plus the initial FM as five controllable input variables, whereby the macronutrients are converted by the initial FM into new biomass, thus defining the growth rate and final FM yield.

Using sucrose concentration in the cultivation medium as a key parameter, a substantial increase in the growth rate and final FM yield of BY-2 cells at the end of the batch phase was achieved through multi-criteria Pareto optimization. Although the mechanistic white-box modeling aims to construct a model precisely representing the collected data, the predictability of conditions not represented in the data is neglected. Thus, the model could not predict cell growth under non-optimal conditions deviating from the parameter space conditions in the experiments used for model setup and refinement (Nausch et al., 2023a). Moreover, such mechanistic white-box models become limited in cases where (i) the relevant process knowledge is missing (e.g., the plant cell metabolism and kinetics); (ii) not all relevant parameters can be measured (e.g., intracellular macronutrient pools); (iii) there is high variation in the data (e.g., between replicates).

In principle, small-scale bioreactors or even microtiter plates can be used to obtain more data. However, in the case of PCC, the data obtained in these systems are hardly transferable to industrial large-scale bioreactors. While suitable scale-down models were already developed for mammalian cells (i.e., CHO), this has not yet been achieved for PCCs due to several reasons: (i) Plant cells (with an average diameter of 100–500 µm) have a volume that is 100 to 1,000 times larger than that of mammalian cells (which typically measure 10–100 µm in diameter) and sediment up to 25 times faster. Therefore, they cannot be regarded as two-phase systems consisting of only a gas and liquid phase, with in the liquid phase homogeneously distributed cells. Instead, PCCs represent a three-phase system comprising a gas, liquid and solid phase, with the plant cells as a solid phase. This introduces an additional dimension of heterogeneity. (ii) PCCs are associated with rheological constraints as the PCC medium represents a viscous, non-Newtonian broth, leading to imperfect mixing. (iii) PCCs are shear sensitive, so the stirrer and mixing speed must be controlled during scale-up to avoid cell damage (Doran, 1999; Kieran et al., 1997; Cheung et al., 2018). This limits the applicability of small-scale bioreactors for process optimization so that they are rather used for process establishment, i.e., cell line generation and selection. For the actual process optimization, larger bioreactors are needed to get representative data. However, the larger the scale, the fewer conditions can be tested (Cheung et al., 2018).

As an alternative to mechanistic white-box modeling, Bayesian experimental design (BED) is a global black-box optimization strategy that does not make any assumptions about the properties of the process. BED represents an adaptive experimental design approach that utilizes Bayesian optimization (BO) to iteratively perform experiments to identify optimal process parameters (Duris et al., 2020; Garnett, 2023; Rainforth et al., 2024; Zhang et al., 2021; Figure 2). A key difference between BED and mechanistic (theory-based) modeling lies in the aim of BED not to create a model that is as accurate as possible but to efficiently find the optimal process settings. In contrast to traditional, non-sequential DoE methods (e.g., one-factor-at-a-time, response surface methodology, full factorial, or partial factorial DoE), BED represents a sequential DoE approach, builds a surrogate model of the objective function, and utilizes an acquisition function for maximizing the informational content of the experiment series. Thus, BED iteratively performs experimental design, execution, and evaluation and adapts the experimental design based on previous experiment results in each iteration. Thereby, BED aims to minimize the total number of experiments for the model setup and process optimization. Moreover, whereas DoE approaches are limited to low-dimensional design spaces with a limited number of discrete parameter levels and manually defined experimental designs (e.g., Bayer et al., 2021), BED allows the exploration of higher-dimensional continuous design spaces guided by the acquisition function that optimizes the informational gain of each experiment. Consequently, BED promises to be a data-driven, exploratory, bias-free, and efficient alternative to traditional DoE that is particularly suited for complex-to-evaluate bioreactor-based fermentation processes with multiple parameters (i.e., batch, fed-batch, semi-continuous, and continuous) at an industrial scale.

**FIGURE 1:**
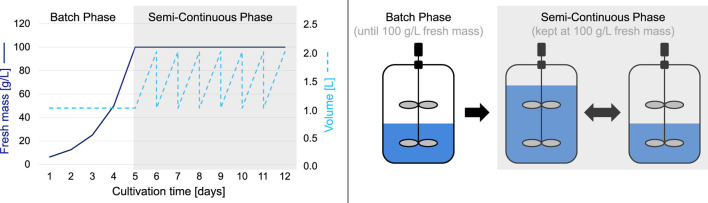
Typical cultivation process of BY-2 cells consisting of an initial 1-week batch phase followed by a multi-week semi-continuous phase.

In this article, we investigate the applicability of BED to PCC fermentation processes of BY-2 and maximize the productivity of the system by means of biomass formation per time while minimizing the resource input and additionally identifying the best trade-off between both. In order to search for an optimal cultivation medium composition that promotes biomass formation (i.e., as a ratio between nutrient and FM input and FM output) during typical batch fermentation of BY-2 cells in STRs (Patent WO2015165583A1, Figure 1), we propose a multi-variate, multi-objective, and batch-wise BED algorithm. With the long-term intention to establish a mechanism for adaptive, dynamic real-time process monitoring and control (e.g., for a semi-continuous long-term fermentation), we focused on FM and sucrose as parameters that can be measured in- or online.

Accordingly, the contributions of our article can be summarized as follows:

1. We propose a novel and data-driven multi-variate, multi-objective, and batch-wise BED approach for yield-optimal parameterization of biomass formation.
2. We apply our proposed BED algorithm for analyzing and finding optimal cultivation medium compositions in the batch fermentation of BY-2 cells in STRs and report the experiment results.
3. We analyze, interpret, and discuss the results in relation to the findings of previous studies (i.e., parameter–parameter and parameter–objective relationships in the BY-2 batch fermentation).

This proof-of-concept study will demonstrate that BED can be used to efficiently optimize a typical batch fermentation process of BY-2 by utilizing parameters measurable in- or online, without aiming for a complete optimization of the fermentation process itself.

## 2 Materials and methods

### 2.1 Cultivation of tobacco cells

As a representative batch fermentation for BY-2 cells, we aimed to optimize the patented process (Patent WO2015165583A1, Figure 1) that employs the Murashige & Skoog (MS) medium, as this is typically used for the cultivation of BY-2 cells (Nausch et al., 2023b). The BY-2 cells were grown as previously described by Nausch et al. (2023a). In summary, the cultivation of BY-2 routine cells was done in 200 mL of MS medium in 1,000-mL Erlenmeyer shake flasks in a Climo-Shaker ISF1-X orbital shaker (Kuhner Shaker, Herzogenrath, Germany) at 26°C and 160 rpm, and the BY-2 cells were passaged every 7 days. For cultivation by fermentation in STRs (Patent WO2015165583A1), these 7-day-old BY-2 cells from the routine culture were used to inoculate 1,000 mL of a defined medium (Supplementary Table S1) in a 2,000-mL STR (Getinge Deutschland, Rastatt, Germany).

The patented cultivation in STRs is divided into two phases (Figure 1). In phase 1, the batch phase, the BY-2 cells are cultivated over several days in a constant medium volume without any additional medium feed until an FM of 100 g/L is reached. The batch phase is followed by the semi-continuous phase, in which new cultivation medium is continuously fed into the bioreactor to keep the FM constant at 100 g/L, the medium is harvested daily, and the volume is reduced to the initial one (in this example, 1,000 mL). Due to significantly different optimization conditions between a batch phase (quasi-discrete experiments with static parameter settings that are independent of a previous state) and a semi-continuous phase (long-term sequence of several interdependent parameter settings with intermediate measurements), a two-stage optimization system is required. Consequently, in this article, we focus solely on the optimization of the batch phase (which is why the semi-continuous phase is grayed out in Figure 1).

### 2.2 Cell biomass and macro nutrient analysis

While FM was determined through vacuum infiltration, the DM was determined by subsequent drying in an oven at 60°C for 3 days (Nausch et al., 2023a). Nutrients were determined by specific offline assays (sucrose: abcam ab83387, ammonium: Sigma-Aldrich MAK310, nitrate: Cayman Cay780001, phosphate: abcam ab65622) according to the manufacturer’s instructions.

### 2.3 Bayesian experimental design (BED)

BO is a sample-efficient statistical method for surrogate-based global optimization of expensive-to-evaluate and noisy objective functions y=f(x)+ε (Greenhill et al., 2020; Kumar et al., 2022). Based on the BO framework, BED represents a sequential DoE method for scientific experimentation in engineering systems. BED is a global optimization approach that does not make any assumptions about the properties of the to-be-optimized system. As a black-box optimization algorithm, BED can be applied (but limited) to black-box systems that (1) do not have a closed-form representation; (2) do not provide functional derivatives; (3) only allow for point-wise evaluation (Greenhill et al., 2020). Under the influence of a noise term ε , the objective function f(x) transforms a vector of n independent input variables $x={\left[x_{1},x_{2},\ldots ,x_{n}\right]}^{T}\in X\subseteq R^{n}$ into m dependent objective variables $y={\left[y_{1},y_{2},\ldots y_{m}\right]}^{T}\in Y\subseteq R^{m}$ . The multi-variate and multi-objective global black-box optimization problem can be expressed as finding the solution to Equation 1:

$$x^{*}=\arg\underset{x\in X}{\max}f\left(x\right)$$ (1)

by optimizing the analytically unknown objective function yielding Equation 2:

$$y^{*}=\underset{x\in X}{\max}f\left(x\right)=f\left(x^{*}\right)$$ (2)

Therefore, the aim of BED is to find the optimal parameterization $x^{*}$ within the n -dimensional design space $X\in R^{n}$ that yields an optimal process output $y^{*}$ in the m -dimensional objective space $Y\in R^{m}$ . For sequential optimization, BED comprises two core components: (1) a surrogate model to model f(x) and (2) an acquisition function α(x) to determine the next experiments. While extra trees, random forests, tree parzen estimators, support vector machines, and Bayesian neural networks are suitable surrogate model options, Gaussian processes (GPs) (also known as Gaussian process regressors) (Rasmussen, 2004) are the preferred surrogate model choice because of their data efficiency, flexibility, simplicity, and built-in quantification of uncertainty (Garnett, 2023; Johnson et al., 2025; Kariminejad et al., 2024; Harris et al., 2024; Kumar et al., 2022; Rodemann et al., 2024). Consequently, GPs are the most commonly used surrogate models in data-sparse, noisy, and low-dimensional optimization problems. GPs are non-parametric and probabilistic models that are constructed directly from a dataset, allowing the complexity of the model to grow with the number of data elements (Duris et al., 2020). GPs fulfill the requirement of the acquisition function to provide uncertainty estimates of the objective function for given parameter sets (Duris et al., 2020). A GP comprises a mean m(x) and a covariance function (also denoted kernel) $k\left(x,x^{\prime }\right)$ (Equation 3). The kernel describes the similarities between two sets of input parameters x and $x^{\prime }$ (Duris et al., 2020).

$$f\left(x\right)∼GP\left(m\left(x\right),k\left(x,x^{\prime }\right)\right)$$ (3)

BED substitutes the optimization of the expensive-to-evaluate objective function with the optimization of an inexpensive, analytically differentiable, and therefore, more tractable acquisition function α:X→R (Garnett, 2023). The acquisition function α(x) uses the expected mean m(x) and uncertainty $k\left(x,x^{\prime }\right)$ of the GP to make an informed decision about the next set of parameter values by assigning a score to each parameter location to maximize the information gain (Gabler and Wollherr, 2022; Garnett, 2023).

**FIGURE 2:**
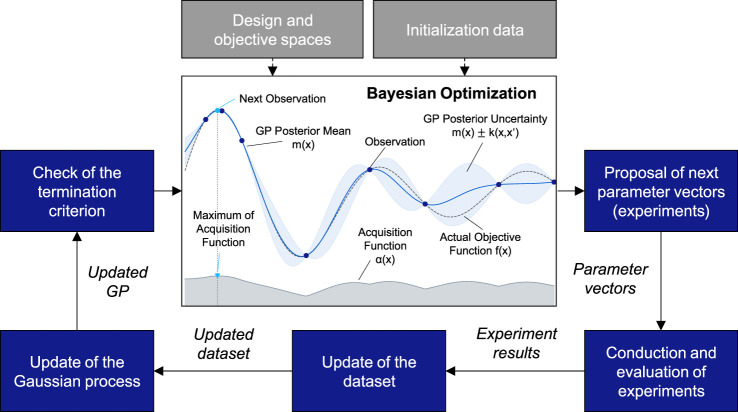
Bayesian experimental design procedure and Bayesian optimization components (based on Al-Hafez, 2021).

According to the BED algorithm (Figure 2), starting with a predefined design space X comprising one or multiple parameters $x_{i}$ and based on an initial dataset D(x,y) , BED iteratively proposes a set of parameter values (e.g., nutrient concentrations) that defines the next set of experiments (step 1). The initial dataset D(x,y) can contain historical process data or can be systematically collected using quasi-random sampling approaches (e.g., Sobol algorithm (Sobol’, 1967) or space-filling designs) that aim to equidistantly cover and explore X . After BED has proposed a parameter vector $x_{t}$ at iteration t , the experiments are conducted and evaluated (step 2). The evaluation (measuring and quantifying the process outcomes for each objective) yields the experiment results $y_{t}$ for each parameter set x (step 3). The results of the experiments are added to the dataset D(x,y) (step 4). The GP surrogate model is then updated (step 5), and afterward, the fulfillment of the termination criterion is checked (step 6). This constitutes the end of the iteration. The termination criterion can be arbitrarily defined by the expert and can include acceptance levels for each objective, experimental budgets, and convergence measures. As long as the termination criterion is not fulfilled and the optimization has not been manually terminated by the expert, experimentation continues.

### 2.4 Multi-variate, multi-objective, and batch-wise Bayesian experimental design for optimization of the BY-2 batch fermentation

**FIGURE 3:**
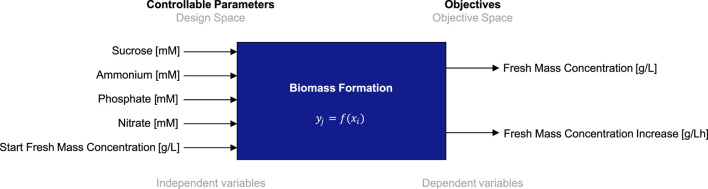
Process scheme with controllable input parameters (independent variables) and objectives (dependent variables) for the biomass formation in a batch fermentation process of BY-2 cells.

To establish BED for the optimization of biomass formation and nutrient consumption of BY-2 in batch cultivation mode, we define a five-dimensional design space comprising the five individual macronutrients of the cultivation medium, that is, sucrose, ammonium, nitrate, and phosphate, and the initial FM concentration. The lower and upper limits for each medium component were determined in previous experiments (Nausch et al., 2023a). Macronutrient concentrations were chosen so that reducing or increasing them further did not enhance the BY-2 growth rate or final biomass yield or even reduced them. Furthermore, we define a two-dimensional target space that comprises the final FM concentration (i.e., biomass) and the corresponding FM concentration increase per hour (i.e., growth rate). The full specifications of the to-be-optimized biomass formation process, including parameters and objectives, are given in Figure 3. Details on the parameters (units and valid value ranges) and objectives (units, minimum and maximum values, and direction of optimization) are contained in Tables 1, 2. As stated above, minimum and maximum values are based on process experience and previous experiments (Nausch et al., 2023a). Accordingly, we define the biomass formation as a multi-variate and multi-objective optimization problem y=f(x) , assuming a black-box system comprising five controllable input parameters and two fully observable objectives (FM concentration (g/L) and FM concentration increase (g/(L∗h)) with $x_{i}$ being the set of input parameters with i∈{sucrose, phosphate, ammonium, nitrate, start FM} and $y_{j}$ being the two objective variables with j∈{final FM, FM increase} .

**TABLE 1:** Overview of controllable input parameters (design space) for biomass formation in a batch fermentation process of BY-2 cells.

Design parameter cultivation medium component	Unit	Minimum value	Maximum value
Sucrose concentration (C_12_H_22_O_11_)	mM	20	175
Ammonium concentration (NH_4_^+^)	mM	5	60
Phosphate concentration (PO_4_^3−^)	mM	2	15
Nitrate concentration (NO_3_^−^)	mM	5	150
Start fresh mass concentration	g/L	5	22

**TABLE 2:** Overview of objectives (objective space) for biomass formation in a batch fermentation process of BY-2 cells.

Target variables	Unit	Minimum value	Maximum value	Optimization type
Fresh mass concentration	g/L	0	200	Maximization
Fresh mass concentration increase	g/(L×h)	0	25	Maximization

We utilize BED as a statistical sequential optimization method that aims to efficiently identify optimal parameter values $x^{*}$ that yield an optimal process outcome $y^{*}$ of the objective function f(x) (Greenhill et al., 2020). Due to the high complexity of fermentation processes in STRs at industrial scales, only a limited number of experiments can be carried out. Given the small dataset size, we decided to use GPs as surrogate models because of their data efficiency. For multi-objective optimization, a multi-objective GP is used to model the two objectives of FM concentration and FM concentration increase (i.e., growth rate). Based on the five input variables, the GP performs a regression task predicting the objective value for each one of the objectives within the corresponding value range. The GP is initialized using a constant mean as the mean function and the Matérn-5/2 kernel as the covariance function. We initialize the GPs on historical data from Nausch et al. (2023a), comprising a total of ten experiments (Batch 0 in Supplementary Tables S2, S3). Due to the duration of individual experiments of 7 days (164–172 h), we decided to run four optimization iterations (termination criterion), followed by two iterations for confirmation of the results. The GP models the input noise as homoscedastic noise. Automatic relevance determination (ARD) is used to iteratively determine the relevance of each parameter and adjust the GP’s length scale parameters iteratively during optimization (Duvenaud, 2014).

Because the fermentation setup in this study allows the simultaneous fermentation of four separate medium compositions, we propose batch-wise BED allowing the simultaneous provision of four experiment sets (batch-size q=4 ). Accordingly, in each iteration (batch), BED proposes a set of four different medium compositions (experiments). In contrast to single-point optimization (q=1) , where the parameter set with the highest acquisition function value is selected, a set of q experiments with the q highest acquisition function values is proposed. Although, the batch size positively correlates with the required number of experiments because parameter sets with suboptimal informational gain are selected (Zhan et al., 2024), we decided to parallelize experimentation across all four STRs to reduce experimentation time by a factor of four. As a multi-objective, noisy, and batch-wise acquisition function *α*(x), q Noisy Expected Hypervolume Improvement (qNEHVI) is used (Daulton et al., 2021). α(x) uses the hypervolume (HV) metric as a multi-objective performance metric and determines next experiment locations based on the approximated improvement of the HV (Audet et al., 2021).

### 2.5 Software implementation, computational resources, and source code

Our approach for multi-variate, multi-objective, and batch-wise BED was implemented using the Python programming language and the Python library BoTorch (Balandat et al., 2019). BoTorch is a BO programming framework that is built on top of PyTorch (Paszke et al., 2019). The source code is available on GitLab (https://gitlab.cc-asp.fraunhofer.de/fraunhofer-ipt/biodapt). Calculations were performed on hardware running Linux Ubuntu 22.04 equipped with Dual Intel Xeon Gold CPUs with 16 cores, Dual Nvidia Quadro RTX5000 with 16 GB, and 384 GB of DDR4 RAM.

## 3 Results and discussion

### 3.1 The BY-2 growth rate can be regulated by nitrate and phosphate, while it is possible to reduce sucrose and ammonium without impacting the growth rate and only affecting the final biomass yield

**FIGURE 4:**
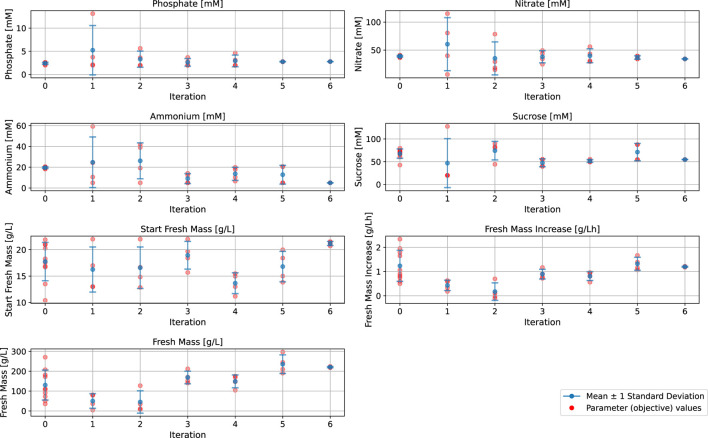
Parameter levels of the input and output parameters for the Bayesian experimental design model setup and refinement for the biomass formation in a batch fermentation process of BY-2 cells. Iterations 1–4: Calibration experiments, Iterations 5–6: Confirmation experiments.

**FIGURE 5:**
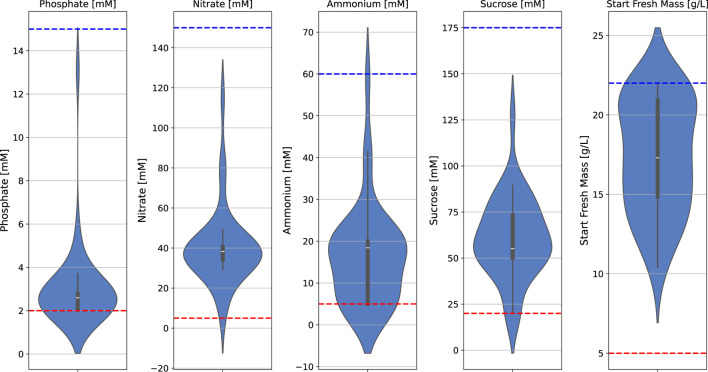
Distribution of all five input parameters after all iterations, including upper (blue) and lower (red) design space limits (dashed lines) in a batch fermentation process of BY-2 cells.

**FIGURE 6:**
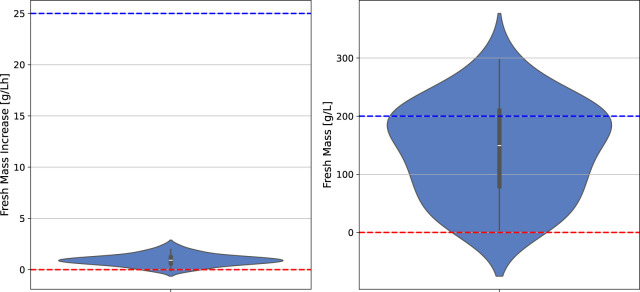
Distribution of all two objective values after all iterations, including upper (blue) and lower (red) objective value limit ranges.

The batch fermentation lasted for 7 days (164–172 h) with daily FM measurements (intervals of 22–26 h). The FM increase was determined for these 7 days (at the end of fermentation) and for 4 days (at the time point at which 100 g/L FM is usually reached and the semi-continuous phase is typically started). The BED algorithm for the optimization of macronutrients and biomass formation (Figure 3; Table 1; Table 2) showed convergence behavior (i.e., stabilization of parameters and objectives over the number of iterations and rapprochement toward consistent values) within the four iterations comprising a total of 16 different media (Figure 4; Supplementary Table S1). The medium composition that led to the highest growth rate and final biomass yield was confirmed in two further iterations. Accordingly, Supplementary Table S1 contains the experimental results, and Figure 4 shows the evolution of the distributions of parameters and objectives along the iterations. In detail, the distributions of all five parameters after all iterations are shown in Figure 5. In analogy, Figure 6 shows the distribution of both FM and FM increase after all iterations. Please refer to the appendix for a detailed analysis of the progressions of parameters and objectives along all iterations (Supplementary Figure S1 (sucrose), Supplementary Figure S2 (ammonium), Supplementary Figure S3 (nitrate), Supplementary Figure S4 (phosphate), Supplementary Figure S5 (start FM), Supplementary Figure S6 (final FM), and Supplementary Figure S7 (FM increase)).

Iteration 0 contains historical data from previous experiments (Nausch et al., 2023a). Iterations 1–4 are the optimization runs, and iterations 5 and 6 are the confirmation runs. The convergence behavior within the first four iterations (iteration 0 not included) is indicated by the narrowing distributions of the values for all four macronutrients (sucrose, ammonium, nitrate, phosphate) and the initial FM (Figure 4). Based on the results of iterations 1 to 4, we selected the set of parameter values that led to the highest final biomass yield (FM) and growth rate (FM increase) while requiring comparatively less concentration of the individual media components (highlighted in blue in Supplementary Table S1). The selected medium was then confirmed in a side-by-side comparison with the standard MS medium used for the cultivation of BY-2 (iteration 5), and we confirmed its reproducibility by parallel fermentations (iteration 6).

During early iterations of the BED algorithm, the starting FM showed the widest spread of the input parameters, and sucrose and ammonium displayed a broader distribution than nitrate and phosphate, which exhibited a strong concentration around specific parameter values (Figure 4). The greater spread of the initial FM, sucrose, and ammonium during the optimization process, compared to nitrate and phosphate, can be interpreted as the BED algorithm is attributing a higher relevance to these parameters, leading to a greater variation compared to those considered less relevant. However, in the case of sucrose and ammonium, the data also indicated that these two parameters can be altered without a substantial impact on the growth rate, although there was a pronounced effect on the final biomass (Figure 6). Vice versa, because of the strong impact of nitrate and phosphate on FM increase (growth rate), these two parameters seem to converge toward specific values and thus can be used to regulate the growth rate.

In analogy to the distributions of the media components (Figure 5), Figure 6 shows the distribution of final FM and growth rate. The final FM exhibits a broader distribution than the growth rate. The wider spreading of final FM might result from the fact that this objective is affected by all five parameters and their corresponding changes, while the growth rate is mainly affected by nitrate and phosphate.

Compared to the MS standard medium, the final medium M31 had a substantially reduced sucrose concentration as carbon source of −38%, which complies with the previous study, in which the cultivation medium was optimized via a mechanistic model for the nutrient consumption, indicating that sucrose is in excess in the MS medium and can be reduced without impacting growth rate and only affecting the final biomass (Nausch et al., 2023a).

Similarly to sucrose, the nitrogen source ammonium was also drastically lowered in the M31 medium by −76%, which in turn complies with the fact that alternative cultivation media such as the Gamborg B5 (Gamborg et al., 1968), Schenk and Hildebrandt (SH) (Schenk and Hildebrandt, 1972), and Chu N6 Chih-ching (Chu et al., 1975) contain only low levels of ammonium at 2.02 mM, 2.61 mM and 7.00 mM, respectively. This might be explained by the fact that BY-2 cannot grow in a medium without ammonium (Holland et al., 2010; Ullisch, 2012), but higher ammonium levels are potentially cytotoxic (Behrend and Mateles, 1975; Behrend and Mateles, 1976). However, as already discussed by Nausch et al. (2023a), another study in which the MS medium was optimized using DoE showed that a reduction of ammonium to 6.87 mM led to a decreased final biomass after a 7-day batch cultivation in shake flasks (Häkkinen et al., 2018). In addition, in two further studies, it has been observed for a similar shake-flask setting that an increase of ammonium up to 51.54 mM promotes the BY-2 final biomass (Holland et al., 2010; Ullisch, 2012; Ullisch et al., 2012; Holland, 2013). Hence, even though lower ammonium concentrations did not seem to affect the growth rate, they did limit the final biomass yield.

In contrast to ammonium, the nitrate level as the second nitrogen source was only slightly altered in the M31 medium at −13%. The reasons for that might be that, on the one hand, nitrate is metabolized to ammonium and thus must compensate for the reduction in ammonium in the medium. On the other hand, an increase in the nitrate concentration negatively impacts the BY-2 growth, as previously shown (Holland et al., 2010; Ullisch, 2012; Ullisch et al., 2012; Holland, 2013; Vasilev et al., 2013). Accordingly, nitrate might be used to adjust the growth rate if needed.

Phosphate remained nearly constant with +2% in the M31 medium, even though it has already been found that increasing phosphate concentration in the MS medium from 2.72 mM to 10.00 mM boosted the biomass accumulation five-fold in a 7-day batch fermentation in shake flasks (Holland, 2013). Hence, adjusting the phosphate might be another option to alter the growth rate in the batch fermentation process.

With respect to the initial FM, the results show that lowering the inoculation FM did not lead to a higher growth rate but rather resulted in a lower final biomass, which contradicts the observation in the previous study (Nausch et al., 2023a). The reason for that must be elucidated. Nevertheless, while the initial conditions yielded a final biomass of up to 81.41 g/L FM and a growth rate of 0.63 g/(L × h), this was increased to 212.41 g/L FM and 1.17 g/(L × h) in the medium M31 in iteration 3.

### 3.2 The BY-2 biomass yield was compromised by the lower sucrose concentration

To confirm the observations from the BED experiments, a side-by-side comparison of both media in a 7-day batch fermentation was conducted (iteration 5), and its reproducibility was confirmed by parallel fermentations (iteration 6).

Notably, there was a high variation between identical runs with both the MS and M31 medium. Such variations are normal and possibly result from the natural variability of the BY-2 cells as a biological component. Nevertheless, the BY-2 growth rates and biomass yields were similar for the MS and M31 media until day 4. Afterward, the BY-2 growth rate declined in the M31 and yielded a substantially lower final biomass at day seven because the sucrose was depleted in the M31 medium (Supplementary Figure S1; Supplementary Table S1). This confirms that a lower sucrose concentration does not impact the growth rate but limits the final FM yield. However, at day 4 to day 5, the FM concentration reached 100 g/L, where typically the semi-continuous phase starts, such as in the historical data used to initialize the BED algorithm, which might explain this mismatch in the optimization. Ammonium was consumed completely within 2 days of fermentation but led to an increased degradation of nitrate without any obvious negative impact on growth rate, which was also true for phosphate, which was consumed within 2 days as well. The latter confirms the previously discussed assumption that BY-2 cells seem to have an intracellular pool for phosphate that is used in case this macronutrient is depleted in the medium (Nausch et al., 2023a). Hence, the final M31 medium yielded similar growth rates to the standard MS medium, while the final biomass yield was lower due to the lower sucrose concentration.

### 3.3 The fresh mass might be used as a surrogate parameter for the biomass for real-time monitoring and optimization of a BY-2 fermentation process

**FIGURE 7:**
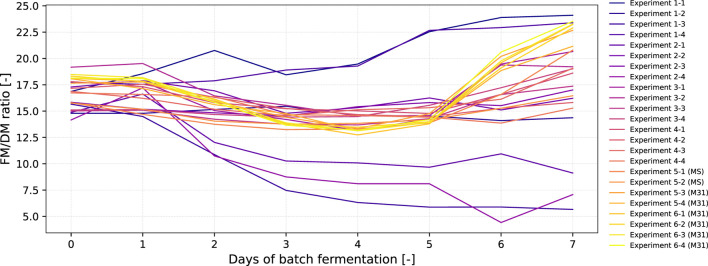
Progression of the FM to DM ratio over the total duration of fermentation (6–7 days) for all 24 fermentations experiments that were conducted within six iterations.

With the long-term intention of establishing a mechanism for adaptive, dynamic real-time process monitoring and control (e.g., for a semi-continuous long-term fermentation), we focused on FM and sucrose as modeling parameters as these can be measured in- or online, allowing adaptive real-time process monitoring and optimization. However, the FM might be an error-prone parameter as it can be impacted by excessive water uptake or loss, while DM represents the actual biomass more accurately. For example, it is well known that when BY-2 cells enter the stationary phase, they stop growing and the DM remains constant, whereas they still consume water so that FM further increases. Thus, we compared the FM/DM ratio in the various runs as shown in Figure 7 and Supplementary Table S2 and Supplementary Table S3.

For most of the iterations in which BY-2 grew (including medium M31), the FM/DM ratio slightly decreased when the cells shifted from the lag to the exponential phase at days 1–3. Starting from days 5–6, this trend changed, and the FM/DM ratio tended to increase, indicating that the cells then entered the stationary phase from the exponential phase. In the case of the MS reference medium, the increase started at days 6–7. This difference might be explained by the fact that the MS medium was designed to enable a typical 7-day shake-flask batch cultivation, while the BED algorithm was trained on historical data, in which the batch fermentation ended at day 4–5 and the semi-continuous phase with feed supplementation started, as mentioned previously. Nevertheless, the FM/DM ratio seems to be rather constant when the BY-2 cells are in the exponential phase (i.e., between the lag and stationary phase), as it is the case in the semi-continuous long-term fermentation of BY-2 cells, in which the FM concentration is kept at 100 g/L FM by feeding (Figure 1). Thus, the FM might be used as a surrogate parameter for biomass for real-time monitoring and optimization of a BY-2 fermentation process.

## 4 Conclusion

The biomass formation of the plant suspension cell line BY-2 in a typical batch fermentation process was successfully analyzed and significantly improved by adapting the nutrient composition in the cultivation medium with only a few experiments and iterations, using a sequential and adaptive BED algorithm, and only using parameters that can be measured in- or online. Notably, the performance equaled that of a previously developed mechanistic white-box model, demonstrating that BED represents an alternative approach for the optimization of biomass formation, particularly in the case when experimental data are limited, which is usually the case for industrial fermentation settings. Our data-driven, black-box BED algorithm allows a bias-free investigation of the experimental design space and thereby facilitates the discovery of novel process insights.

Specifically, our results indicate that nitrate and phosphate can be utilized to regulate the growth rate (up to 40 g/L ⋅ d), whereas sucrose and ammonium can be reduced without affecting the growth rate, though this may influence the final biomass yield (up to 300 g/L FM). Notably, when comparing the concentration of the four macronutrients in the MS medium plus the standard initial FM to the one used in the final M31 medium with the reduced initial FM, the optimized setting led to a 36% increase in the biomass yield by means of a ratio between macronutrient and FM input to FM output.

Importantly, this study aimed to provide the proof-of-concept that BED can be used to optimize typical fermentation processes without aiming to fully optimize the fermentation process itself. Hence, we did not fully analyze the fermentation process to resolve the trade-off between growth rate and final biomass yield. For example, the impact of reduced sucrose and ammonium concentrations on the final biomass yield and growth rate has to be elaborated in the future, as the final biomass yield was compromised through the optimization rounds. Moreover, we will conduct further experiment series using our BED algorithm to analyze its optimization behavior and robustness.

Beyond, we focused on the quasi-discrete optimization of the batch phase. Given the state-dependent and long-term characteristics of the subsequent semi-continuous fermentation phase, regular BED does not represent a suitable approach. In future work, we will therefore investigate contextual BO (Char et al., 2019; Fiducioso et al., 2019) for the optimization of the semi-continuous phase. Moreover, to make BED more efficient and to strengthen acceptance of BED among biopharmaceutical experts, our research will focus on intensifying the collaboration between algorithm and domain experts to investigate how domain knowledge can be integrated into the BED workflow before, during, and after optimization (Kanarik et al., 2023; Hvarfner et al., 2022; Savage and Del Chanona, 2023). To further enhance the overall experimentation, the relationship between batch size and cost-to-target must be analyzed as well.

Nevertheless, we demonstrated the applicability of data-driven BED for data-efficient non-sequential experimental design for the plant cell line BY-2. BED might also be applicable to microbial and mammalian cell lines in batch fermentation mode.

## Data Availability

The data presented in this study is available on GitLab: https://gitlab.cc-asp.fraunhofer.de/fraunhofer-ipt/biodapt.
