# Supplementary material for: Bayesian experimental design for optimizing medium composition and biomass formation of tobacco BY-2 cell suspension cultures in stirred-tank bioreactors
Source: Front Bioeng Biotechnol. 2025 Sep 19;13:1617319. doi: 10.3389/fbioe.2025.1617319 (PMC12492029; doi:10.3389/fbioe.2025.1617319)
Supplement: Supplementary file 1 [file DataSheet1.pdf]

Supplementary Material

1 SUPPLEMENTARY DATA

Experiment Number	Batch (Iteration)	ID (Iteration - Experiment)	Input Parameters					Objectives	
			Sucrose	Ammonium	Nitrate	Phosphate	Start FM	Final FM	FM increase
			[mM]	[mM]	[mM]	[mM]	[g/L]	[g/L]	[g/(h*L)]
1	0	0-1	72.87	19.17	40.60	2.59	20.98	35.30	0.50
2	0	0-2	68.97	20.36	39.41	1.97	21.00	91.78	0.60
3	0	0-3	69.35	19.95	39.84	2.46	18.28	107.50	0.82
4	0	0-4	79.69	19.68	40.58	2.40	10.40	73.00	0.91
5	0	0-5	70.49	19.64	39.59	2.54	20.28	111.30	1.06
6	0	0-6	73.23	19.88	36.35	2.60	17.60	51.10	0.75
7	0	0-7	74.65	18.25	38.18	2.48	21.90	270.80	2.34
8	0	0-8	65.64	19.98	38.02	2.50	13.50	181.70	1.79
9	0	0-9	60.35	18.59	38.82	2.11	16.90	207.90	1.96
10	0	0-10	42.67	20.53	38.23	2.49	16.70	172.20	1.65
11	1	1-1	20.00	10.56	39.96	1.97	13.00	78.55	0.58
12	1	1-2	20.00	59.26	80.51	2.12	13.00	36.36	0.33
13	1	1-3	127.61	5.00	115.04	13.13	22.00	4.13	0.18
14	1	1-4	20.00	24.24	6.44	3.72	17.00	81.41	0.63
15	2	2-1	82.61	38.96	18.37	1.97	14.83	11.67	-0.02
16	2	2-2	44.29	19.20	29.19	5.63	12.85	127.33	0.70
17	2	2-3	80.76	5.00	78.44	1.97	16.68	34.70	0.11
18	2	2-4	89.51	41.52	14.84	3.62	22.00	6.65	-0.10
**19**	**3**	**3-1**	**54.76**	**5.00**	**34.18**	**2.78**	**18.42**	**212.18**	**1.17**
20	3	3-2	41.23	5.00	24.65	1.97	22.00	151.60	0.78
21	3	3-3	55.46	12.13	43.80	3.69	15.69	170.60	0.91
22	3	3-4	39.30	14.16	49.28	1.97	19.69	140.54	0.72
23	4	4-1	49.24	10.26	43.27	1.97	15.48	175.70	0.96
24	4	4-2	52.44	17.80	29.17	1.97	11.17	104.23	0.56
25	4	4-3	49.49	19.92	56.06	3.11	14.96	169.33	0.93
26	4	4-4	55.59	6.60	31.08	4.64	13.00	146.98	0.80
27	5	5-1	87.64	20.61	39.41	2.72	20.00	297.32	1.67
28	5	5-2	87.64	20.61	39.41	2.72	15.00	244.75	1.39
29	5	5-3	54.76	5.00	34.18	2.78	18.42	209.95	1.16
30	5	5-4	54.76	5.00	34.18	2.78	13.82	190.27	1.07
31	6	6-1	54.76	5.00	34.18	2.78	21.55	222.59	1.21
32	6	6-2	54.76	5.00	34.18	2.78	21.43	217.75	1.18
33	6	6-3	54.76	5.00	34.18	2.78	20.70	218.97	1.19
34	6	6-4	54.76	5.00	34.18	2.78	21.04	224.03	1.22

Table S1. Parameter and objective values of all experimental data: Iteration 0: input parameter and objective levels of previous experiments ?. Iteration 1 – 4: input parameters and objectives of the Bayesian Experimental Design experiments. Iteration 5: Verification of the final M31 medium by side-to-side comparison with the standard MS medium. Iteration 6: Verification of the final M31 medium by replicates.

Batch (Iteration)	Experiment	Day [-]	FM [g/L]	DM [g/L]	FM/DM ratio [-]
0	1 (MS)	0	20.98	-	-
		1	27.34	-	-
		2	25.80	-	-
		3	35.30	-	-
		4	-	-	-
		5	-	-	-
		6	-	-	-
		7	-	-	-
0	2 (MS)	0	21.00	-	-
		1	21.70	-	-
		2	27.40	-	-
		3	42.40	-	-
		4	61.69	-	-
		5	91.78	-	-
		6	-	-	-
		7	-	-	-
0	3 (MS)	0	18.28	1.00	18.28
		1	21.80	1.20	18.17
		2	29.30	1.96	14.95
		3	45.30	3.06	14.80
		4	69.80	4.73	14.76
		5	107.50	6.89	15.60
		6	-	-	-
		7	-	-	-
0	4 (MS)	0	10.40	0.58	17.93
		1	16.11	1.31	12.30
		2	25.08	1.78	14.09
		3	42.00	2.56	16.41
		4	73.00	4.58	15.94
		5	-	-	-
		6	-	-	-
		7	-	-	-
0	5 (MS)	0	20.28	0.68	29.82
		1	29.28	1.08	27.11
		2	43.52	2.28	19.09
		3	70.56	3.96	17.82
		4	111.30	6.50	17.12
		5	-	-	-
		6	-	-	-
		7	-	-	-
0	6 (MS)	0	17.60	0.70	25.14
		1	21.90	0.50	43.80
		2	21.50	0.80	26.88
		3	31.30	2.10	14.90
		4	51.10	3.50	14.60
		5	-	-	-
		6	-	-	-
		7	-	-	-
0	7 (MS)	0	21.90	1.25	17.52
		1	-	-	-
		2	-	-	-
		3	83.30	5.35	15.57
		4	130.10	8.31	15.66
		5	185.00	10.98	16.85
		6	270.80	13.76	19.68
		7	-	-	-
0	8	0	13.50	0.56	24.11
		1	-	-	-
		2	-	-	-
		3	51.80	3.15	16.44
		4	79.50	5.04	15.77
		5	122.60	7.49	16.37
		6	181.70	10.04	18.10
		7	-	-	-
0	9	0	16.90	0.80	21.13
		1	-	-	-
		2	-	-	-
		3	63.10	3.74	16.87
		4	100.40	6.31	15.91
		5	146.30	8.69	16.84
		6	207.90	10.88	19.11
		7	-	-	-
0	10	0	16.70	0.92	18.15
		1	-	-	-
		2	-	-	-
		3	62.00	3.51	17.66
		4	100.80	6.18	16.31
		5	142.60	8.20	17.39
		6	172.20	7.92	21.74
		7	-	-	-
1	11	0	11.30	0.67	16.87
		1	18.18	0.98	18.55
		2	28.03	1.35	20.76
		3	40.97	2.22	18.45
		4	54.32	2.79	19.47
		5	70.56	3.13	22.54
		6	76.20	3.19	23.89
		7	78.55	3.26	24.10
1	12	0	10.50	0.71	14.79
		1	14.79	1.00	14.79
		2	20.46	1.35	15.16
		3	23.66	1.53	15.46
		4	24.04	1.65	14.57
		5	27.60	1.90	14.53
		6	30.87	2.19	14.10
		7	36.36	2.53	14.37
1	13	0	25.69	1.64	15.66
		1	18.40	1.27	14.49
		2	11.30	1.04	10.87
		3	7.25	0.97	7.47
		4	5.56	0.88	6.32
		5	4.59	0.78	5.88
		6	4.36	0.74	5.89
		7	4.13	0.73	5.66
1	14	0	14.90	0.84	17.74
		1	21.40	1.22	17.54
		2	29.15	1.63	17.88
		3	44.22	2.34	18.90
		4	59.76	3.10	19.28
		5	74.58	3.29	22.67
		6	78.89	3.44	22.93
		7	81.41	3.48	23.39
2	15	0	15.62	1.05	14.88
		1	13.46	0.81	16.62
		2	11.67	0.97	12.03
		3	12.72	1.24	10.26
		4	11.79	1.17	10.08
		5	12.86	1.33	9.67
		6	12.25	1.12	10.94
		7	11.67	1.28	9.12
2	16	0	10.89	0.63	17.29
		1	15.56	0.87	17.89
		2	17.10	1.01	16.93
		3	29.29	1.98	14.79
		4	45.38	2.96	15.33
		5	69.08	4.25	16.25
		6	102.32	6.77	15.11
		7	127.33	7.85	16.22

Table S2. FM, DM, and FM/DM ratio data measured and calculated daily over the course of six iterations comprising a total of 34 fermentation runs (Part 1: experiments 1 - 16)

Batch (Iteration)	Experiment	Day [-]	FM [g/L]	DM [g/L]	FM/DM ratio [-]
2	17	0	16.00	1.01	15.84
		1	22.32	1.47	15.18
		2	26.42	1.78	14.84
		3	28.60	1.97	14.54
		4	29.92	1.94	15.42
		5	30.82	1.95	15.81
		6	31.20	2.01	15.52
		7	34.70	2.04	17.01
2	18	0	23.50	1.66	14.16
		1	18.59	1.08	17.21
		2	13.32	1.24	10.74
		3	9.63	1.10	8.75
		4	7.86	0.97	8.10
		5	8.91	1.10	8.10
		6	3.76	0.85	4.42
		7	6.65	0.94	7.07
3	19	0	18.89	1.07	17.65
		1	24.57	1.38	17.80
		2	34.92	2.32	15.05
		3	52.58	3.70	14.21
		4	75.60	5.75	13.15
		5	113.77	7.97	14.27
		6	194.85	10.01	19.47
		7	212.18	10.27	20.66
3	20	0	26.45	1.38	19.17
		1	32.40	1.66	19.52
		2	41.29	2.51	16.45
		3	59.39	3.82	15.55
		4	85.05	5.85	14.54
		5	102.47	7.03	14.58
		6	148.06	7.64	19.38
		7	151.60	7.89	19.21
3	21	0	13.06	0.87	15.01
		1	16.15	1.07	15.09
		2	24.19	1.70	14.23
		3	36.98	2.69	13.75
		4	56.01	4.09	13.69
		5	95.42	6.68	14.28
		6	147.10	8.88	16.57
		7	170.60	9.82	17.37
3	22	0	17.22	1.14	15.11
		1	21.97	1.45	15.15
		2	29.36	2.00	14.68
		3	43.71	3.03	14.43
		4	60.80	4.20	14.48
		5	88.66	5.70	15.55
		6	123.64	7.18	17.22
		7	140.54	7.39	19.02
4	23	0	14.60	0.82	17.80
		1	19.40	1.12	17.32
		2	27.50	1.74	15.80
		3	41.42	2.72	15.23
		4	65.35	4.35	15.02
		5	105.95	7.17	14.78
		6	149.99	9.04	16.59
		7	175.70	9.44	18.61
4	24	0	9.95	0.58	17.16
		1	13.51	0.77	17.55
		2	17.61	1.08	16.31
		3	20.89	1.37	15.25
		4	31.83	2.17	14.67
		5	48.77	3.37	14.47
		6	76.64	5.02	15.27
		7	104.23	6.59	15.82
4	25	0	14.00	0.83	16.87
		1	19.63	1.21	16.22
		2	26.42	1.74	15.18
		3	37.87	2.60	14.57
		4	60.50	4.00	15.13
		5	88.10	5.75	15.32
		6	136.00	8.43	16.13
		7	169.33	8.88	19.07

Batch (Iteration)	Experiment	Day [-]	FM [g/L]	DM [g/L]	FM/DM ratio [-]
4	26	0	13.05	0.78	16.73
		1	15.25	0.92	16.58
		2	21.44	1.31	16.37
		3	29.47	2.01	14.66
		4	48.42	3.32	14.58
		5	75.00	5.20	14.42
		6	107.44	7.75	13.86
		7	146.98	9.62	15.28
5	27 (MS)	0	20.70	1.31	15.80
		1	26.30	1.79	14.69
		2	36.99	2.69	13.75
		3	53.34	4.03	13.24
		4	88.74	6.67	13.30
		5	148.82	10.72	13.88
		6	261.53	15.77	16.58
		7	297.32	14.30	20.79
5	28 (MS)	0	15.13	0.96	15.76
		1	20.04	1.32	15.18
		2	27.12	1.93	14.05
		3	38.40	2.79	13.76
		4	68.11	4.92	13.84
		5	105.00	7.41	14.17
		6	195.90	12.83	15.27
		7	244.75	14.87	16.46
5	29 (M31)	0	17.30	0.98	17.65
		1	26.84	1.51	17.77
		2	38.05	2.39	15.92
		3	57.08	3.96	14.41
		4	86.32	6.47	13.34
		5	124.90	8.46	14.76
		6	201.21	9.96	20.20
		7	209.95	9.26	22.67
5	30 (M31)	0	15.90	0.88	18.07
		1	21.43	1.25	17.14
		2	29.03	1.86	15.61
		3	45.95	3.11	14.77
		4	71.88	5.46	13.16
		5	108.98	7.83	13.92
		6	178.17	9.45	18.85
		7	190.27	8.99	21.16
6	31 (M31)	0	21.55	1.19	18.11
		1	29.10	1.63	17.85
		2	40.35	2.50	16.14
		3	67.36	4.87	13.83
		4	84.99	6.67	12.74
		5	122.77	8.90	13.79
		6	209.39	10.69	19.59
		7	222.59	9.58	23.23
6	32 (M31)	0	21.43	1.17	18.32
		1	29.40	1.65	17.82
		2	39.10	2.47	15.83
		3	60.46	4.39	13.77
		4	85.82	6.37	13.47
		5	111.24	7.95	13.99
		6	204.74	10.72	19.10
		7	217.75	9.51	22.90
6	33 (M31)	0	20.70	1.12	18.48
		1	30.55	1.68	18.18
		2	41.74	2.60	16.05
		3	63.15	4.55	13.88
		4	82.20	6.26	13.13
		5	116.49	8.37	13.92
		6	206.05	10.42	19.77
		7	218.97	9.41	23.27
6	34 (M31)	0	21.04	1.16	18.14
		1	31.10	1.72	18.08
		2	39.90	2.52	15.83
		3	63.09	4.62	13.66
		4	83.07	6.30	13.19
		5	125.46	8.84	14.19
		6	206.84	10.04	20.60
		7	224.03	9.52	23.53

Table S3. FM, DM, and FM/DM ratio data measured and calculated daily over the course of six iterations comprising a total of 34 fermentation runs (Part 2: experiments 17 - 34)

2 SUPPLEMENTARY TABLES AND FIGURES

2.1 Figures

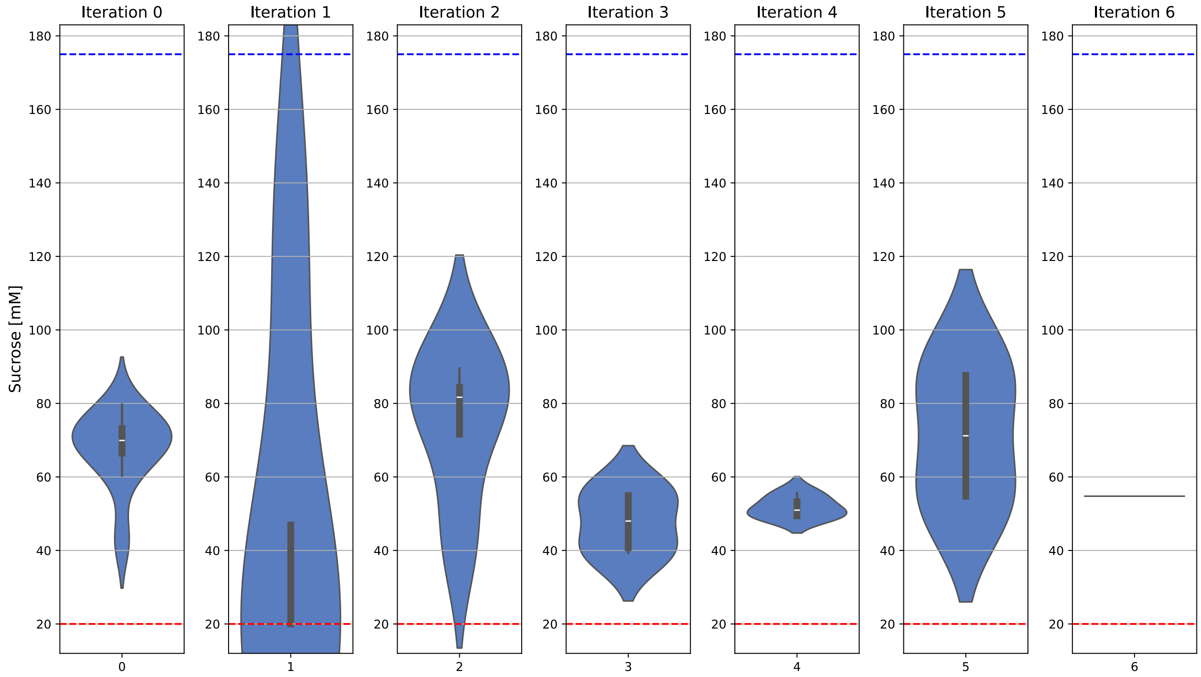

Figure S1. Progression of parameter distributions along the six iterations for sucrose

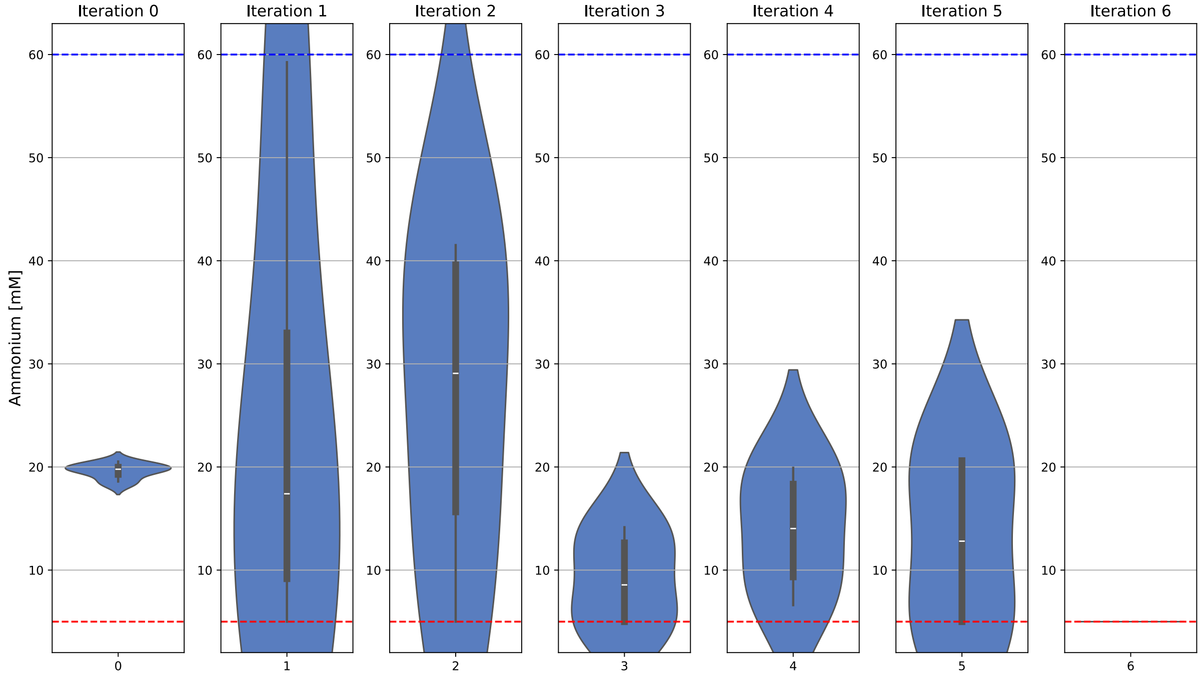

Figure S2. Progression of parameter distributions along the six iterations for ammonium

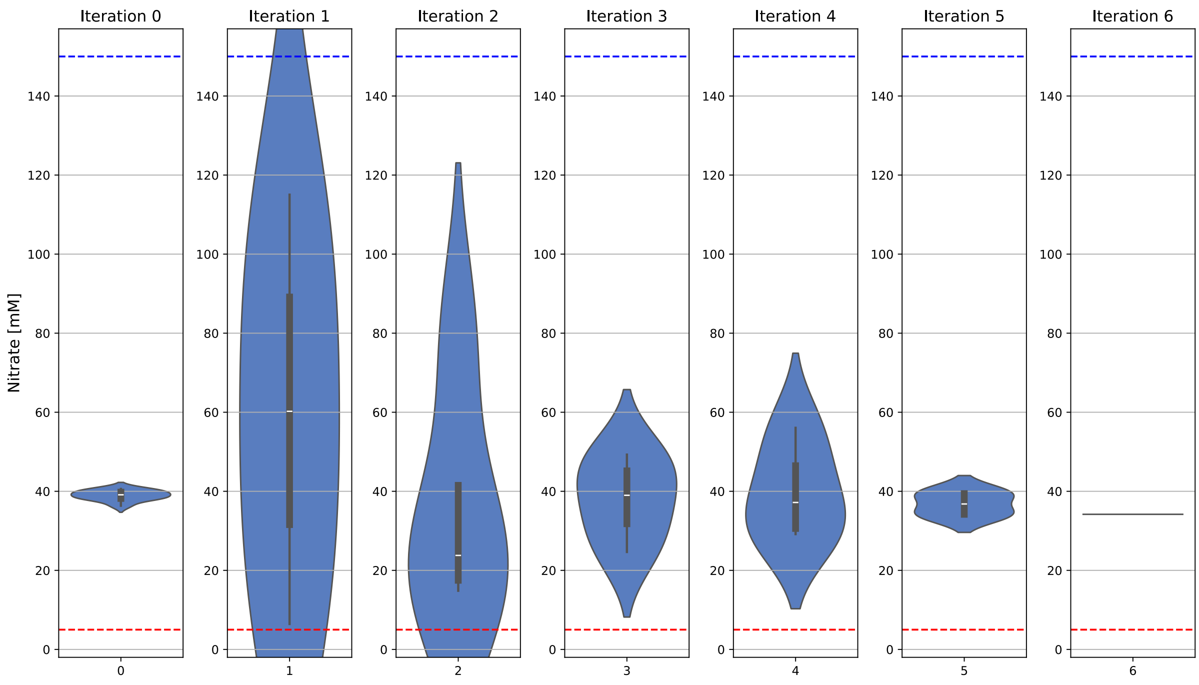

Figure S3. Progression of parameter distributions along the six iterations for nitrate

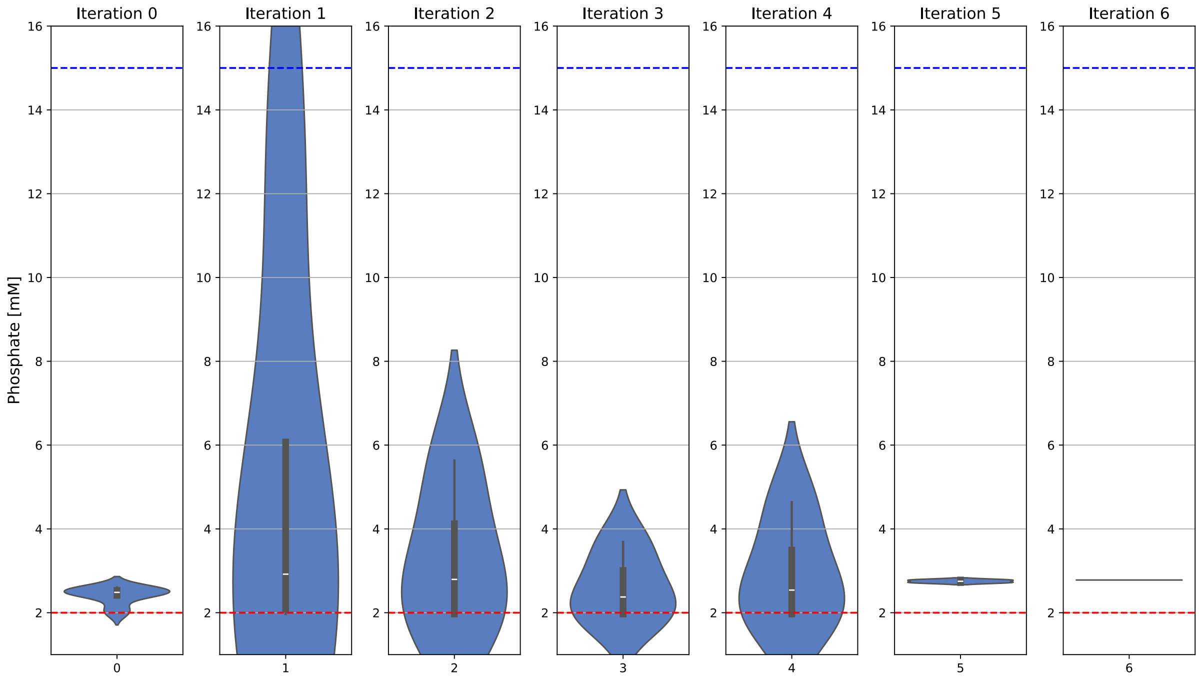

Figure S4. Progression of parameter distributions along the six iterations for phosphate

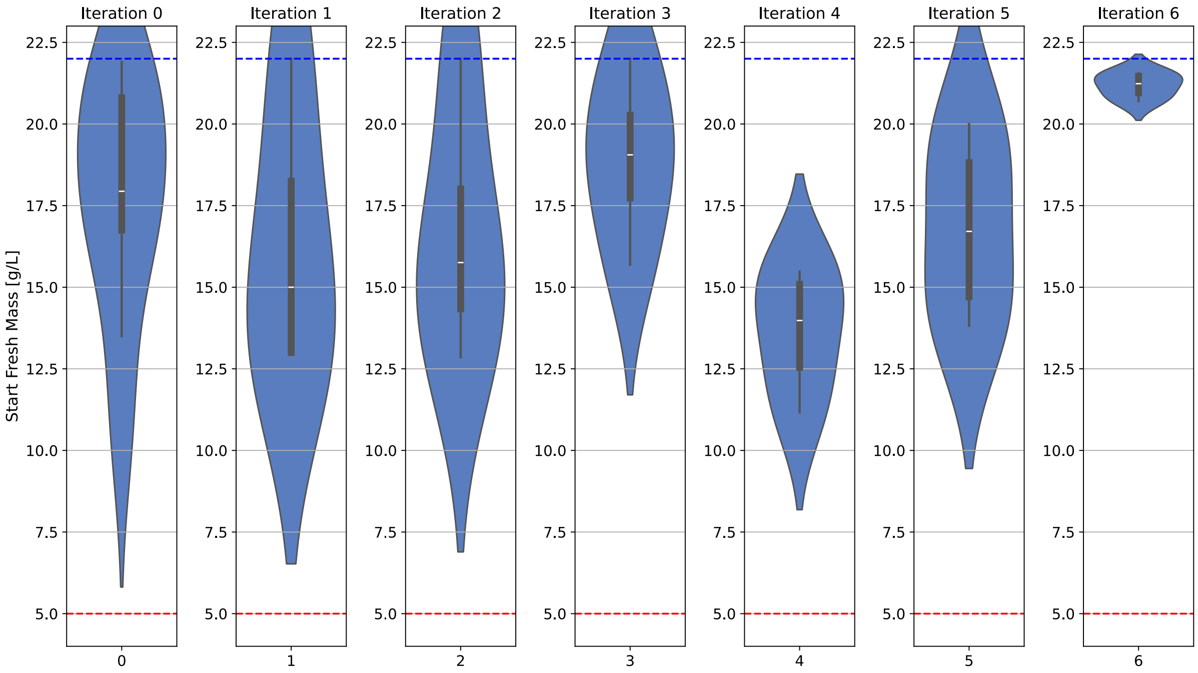

Figure S5. Progression of parameter distributions along the six iterations for start fresh mass

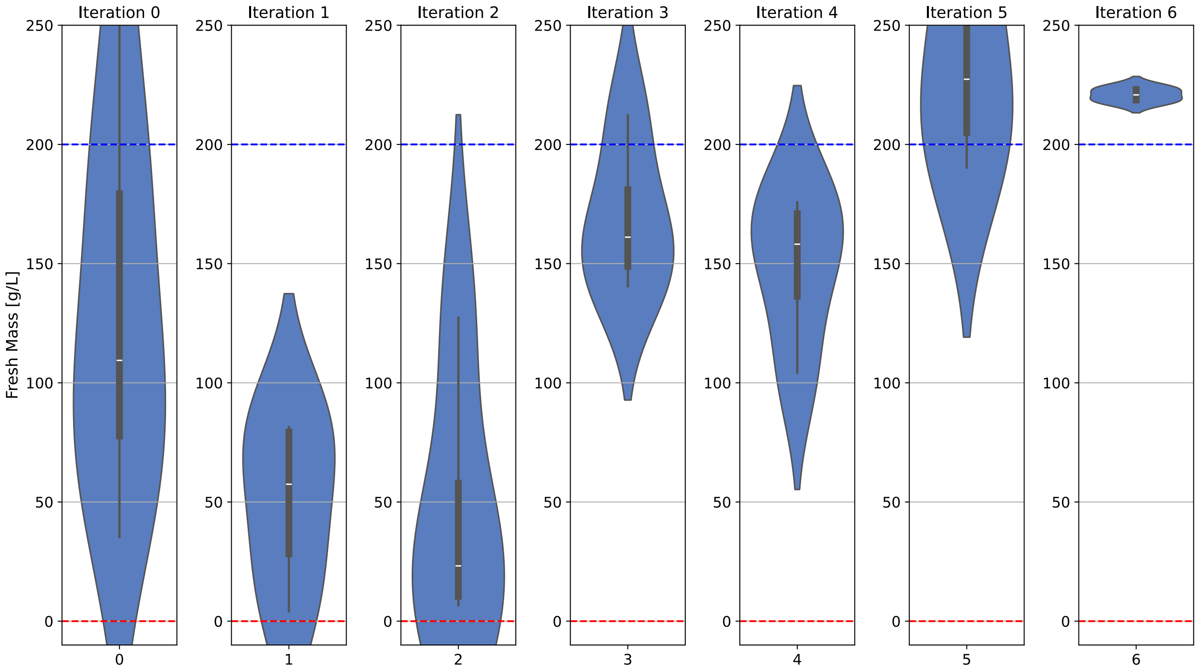

Figure S6. Progression of objective distributions along the six iterations for fresh mass (FM)

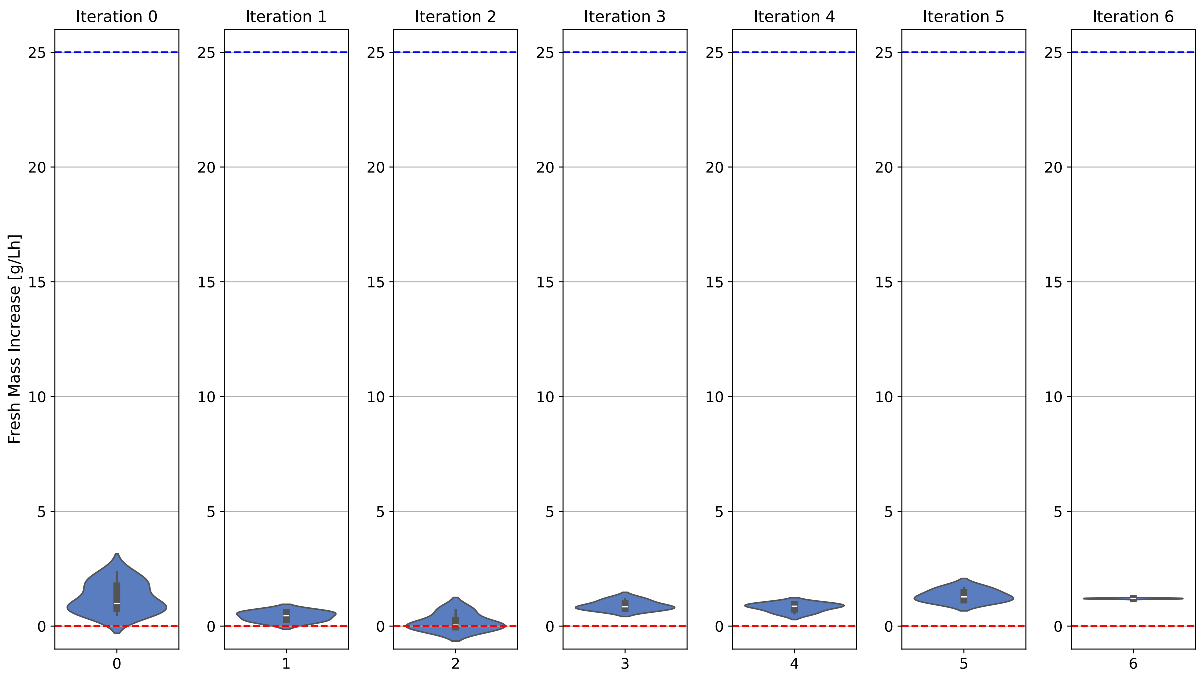

Figure S7. Progression of objective distributions along the six iterations for fresh mass increase (growth rate)
